# The role and value of counsellors in the treatment journeys of people with tuberculosis and their families: Qualitative insights from the South Fly District of Papua New Guinea

**DOI:** 10.1371/journal.pgph.0002572

**Published:** 2024-10-21

**Authors:** Paula Jops, John Cowan, Richard Nake Trumb, Martha Kupul, Allan Kuma, Stephen Bell, Tess Keam, Mathias Bauri, Herolyn Nindil, Suman S. Majumdar, Stacia Finch, William Pomat, Ben J. Marais, Guy B. Marks, John Kaldor, Andrew Vallely, Stephen M. Graham, Angela Kelly-Hanku

**Affiliations:** 1 Kirby Institute, UNSW Sydney, Sydney, Australia; 2 Papua New Guinea Institute of Medical Research, Goroka, Papua New Guinea; 3 Burnet Institute, Melbourne, Australia; 4 Western Provincial Health Authority, Daru, Papua New Guinea; 5 National Department of Health, National TB Program, Port Moresby, Papua New Guinea; 6 Sydney Infectious Diseases Institute (Sydney ID), University of Sydney, Sydney, Australia; 7 Woolcock Institute of Medical Research, Sydney, Australia; 8 Faculty of Medicine and Health, UNSW Sydney, Sydney, Australia; 9 University of Melbourne Department of Paediatrics and Murdoch Children’s Research Institute, Royal Children’s Hospital, Melbourne, Australia; St John's National Academy of Health Sciences, INDIA

## Abstract

Combined education and counselling can contribute to person-centred care for tuberculosis (TB), improving uptake, adherence, and outcomes of treatment for TB disease and TB infection. Though strongly recommended by the World Health Organization for all people diagnosed with TB, education and counselling is not widely implemented in TB programs around the world. In 2016, a pilot TB education and counselling program, delivered by trained professionals and peers, was initiated to support people on TB treatment in the South Fly District of Papua New Guinea. This article reports on select findings from a qualitative study that examined the socio-cultural dimensions of TB, including treatment support such as education and counselling, in the South Fly District. An assessment on data collected during 128 semi-structured in-depth interviews of the role of counsellors on TB treatment journeys revealed strong participant support for the counsellors and the services they delivered, with particular emphasis on the emotional support provided to address fears and concerns related to TB diagnosis and treatment, and to support treatment adherence; valuable attributes of counsellors; their role as intermediaries between patients and health workers; their provision of biomedical knowledge of TB transmission and disease; and their assistance in addressing stigma and discrimination from family and community. Participants also noted how tackling the socio-structural issues that drive TB transmission in people’s homes and communities were beyond the remit of counsellors’ work. TB education and counselling should be an essential part of all TB services to provide support and encouragement for people to continue treatment to completion.

## Introduction

The treatment of tuberculosis (TB) is a lengthy and demanding process, especially for people with multidrug-resistant TB (MDR-TB). Daily medicine must be taken for 4–6 months for drug-sensitive TB (DS-TB), and 6–18 months for MDR-TB. The main concerns for people who are not able to adhere to and complete TB treatment are treatment failure, disease relapse, potential drug resistance amplification, ongoing TB spread within their community, or death [1].

The development and implementation of shorter, safer and better tolerated treatment options for TB disease or infection, is associated with improved adherence and better outcomes [2]. Care and support for adherence is also an integral component of TB service provision and recommended by the World Health Organization (WHO) [3]. However, access to comprehensive TB support services, especially for MDR-TB, may not be widely available for people with TB living in low- and middle-income countries or rural settings, often requiring physical relocation away from home because health service options are limited to urban areas [4]. Other factors affecting TB treatment adherence include low TB knowledge, misinformed treatment beliefs, stigmatisation, availability of social and familial support, the financial burden of treatment, psychological distress associated with anxiety and depression, drug-related adverse effects, and symptom improvement after starting treatment [5–7]. In such instances, education and counselling plays a critical role in ensuring TB treatment completion.

The WHO’s End TB Strategy [8] advocates patient-centred care, which requires a holistic focus on the person with TB, and takes into account their individual needs and circumstances (e.g. social, financial, emotional, and physical), treats them with dignity and respect, and allows them to feel empowered in their decision-making [9]. Education and counselling is an important intervention that can enhance patient- and person-centred care and is strongly recommended by the WHO for all people on TB treatment [10–12]. According to the WHO [12 p.17] “the goal of health education is to provide accurate information so that patients have the information to make the best choice for themselves” …and “While health education aims to equip people with the right knowledge, counselling helps them to apply that knowledge by changing their attitude and behaviour.” The overarching aim of TB education and counselling, no matter the setting, is to ensure that people with TB are supported to make informed choices and actively participate in their own health care [13]. Studies have evidenced the positive impact of education [14,15] and counselling [16,17] separately on treatment adherence for TB disease. Additionally, education and counselling combined can have a positive impact on treatment adherence for TB disease [7,18,19], and can potentially improve outcomes of people receiving TB preventive treatment for TB infection [19–21].

In Daru, South Fly District, Papua New Guinea (PNG), a high-transmission setting for TB and MDR-TB, health promotion and social mobilisation activities focused on enhancing TB detection and treatment support were introduced in 2011. A peer education and counselling model was introduced in 2016. This article examines how counsellors enhanced the treatment experience of people on treatment for TB disease and for caregivers of children on TB preventive treatment from 2019 to 2020.

## Methods

### Study setting

PNG is a lower to middle income country where estimated TB incidence rates are among the highest in the world (424/100,000 population per annum) [22]. TB incidence is noticeably higher in some areas of the country, including the South Fly District, Western Province [23]. In 2014, the PNG National Department of Health activated an emergency multi-sector response due to an ongoing outbreak of MDR-TB on Daru Island, the provincial capital of South Fly District; [24]. In 2016, the model of care decentralised from a facility-based to a community-based model of treatment and care. Six ‘Daru Accelerated Response to Tuberculosis’ (DART) sites were established across Daru Island to provide treatment support. These community treatment sites provide medication administration and meals. While the outbreak has been stabilised [24], the risk of TB transmission remains high [21]. The small island of Daru comprises a mere 14.7 km^2^ and people residing here experience severe overcrowding, undernutrition and poverty [24,25], all factors conducive to TB transmission [26]. We have described the social and geographical setting, along with factors contributing to TB transmission in Daru, elsewhere [25]. TB programmatic interventions in Daru between 2014–2018 have also previously been described elsewhere [24].

An education and counselling initiative to provide person-centred care to people on treatment and their families at Daru General Hospital and DART sites is a key component of the TB treatment program [19]. Current PNG policy is to treat DS-TB for 6 months and MDR-TB for between 9 and 18 months. To enhance support for people with MDR-TB on TB treatment in the South Fly District, a pilot model of TB peer counselling was initiated in Daru in 2016. The peer counsellors, known as PALS (People Affected by, Living with, or Survived TB), received training in education, counselling techniques, TB knowledge, self-care, and topics related to disability and gender-based violence [19]. In 2017 the program expanded to include education and counselling for people with DS-TB and caregivers of children under five years on TB preventive treatment [21]. Education tools were developed and tested to fit the cultural and contextual needs of the setting. All people on treatment were offered education and counselling. For DR-TB, they were offered a minimum of 6 sessions, depending on their individual needs. For DS-TB, it was 3 sessions. However, it should be noted that counsellors were located at the DART sites every day, so if a person on treatment had an issue, they could walk up to a counsellor and have a conversation. This allowed for relationship-building and familiarity over time.

At the time of our study, the team comprised two professional or “lead” counsellors and six peer counsellors mentored by the project counsellor and social worker. People on treatment met with peer counsellors in the first instance. If the case was complex and/or they required additional support, the peer counsellor could refer people to the lead counsellor. Lead counsellors have significant experience in counselling and training, along with an educational degree or qualification in this area.

### Data collection

The findings presented in this article are one component of a larger qualitative study that explored the sociocultural dimensions of TB in the South Fly District of PNG [25,27]. We adopted an interpretive qualitative approach to centre the emic perspectives and lived experience of people with TB on treatment and/or caregivers and family members of people (including children) on treatment.

In this article we report on data collected from 128 semi-structured interviews that explored participants’ perspectives and experiences of TB services and care in Daru, service accessibility and acceptability, and factors contributing to the spread of TB. This current analysis examines their experiences with the education and counselling program.

Participant recruitment and data collection took place on Daru Island, Abam (mainland) and Katatai (mainland) between July 2019-July 2020. Iterative, purposive snowball sampling was used to recruit participants and ensure inclusion of a wide range of participant perspectives, including people on TB treatment and TB preventive treatment, community members, and health service providers. The full sample is described in Table 1. Recruitment was initiated through key informants working in outpatient and inpatient medical services in the study sites, who were directly invited to participate in the study. People who were then approached by the key informants were asked to approach the research team directly either in person at the clinic or in the community, or by sending a free ‘Please Call’ message to the study mobile phone.

**Table 1:** Participant sample.

Participant type	No. participants
**People on treatment for TB** • Adults on treatment for DS TB • Adults on treatment for MDR TB • Person living with HIV on treatment for DS-TB • Person living with HIV on treatment for MDR-TB • Adolescents and young adults on treatment for DS-TB • Adolescents and young adults on treatment for MDR-TB • People who have defaulted or loss to follow up- DS-TB • People who have defaulted or loss to follow up- MDR-TB • People who are being retreated for TB	**52** 10 10 4 5 3 9 1 4 6
Family members of person on treatment for TB* • Person on treatment is a child • Person on treatment is an adolescent • Person on treatment is an adult	**18** 11 2 5
**Caregivers of young (<5 years) children eligible for TB preventive treatment** • Child receiving TB preventive treatment • Child not receiving/refused TB preventive treatment	**16** 10 6
**Key Informants** • Former TB* patients • Healthcare workers • Policymakers and advisors • Program Implementers • Community and church leaders • Non-TB program partners	**42** 6 7 6 16 3 4

*Treated for DS or MDR TB. TB: Tuberculosis; DS: Drug-susceptible; MDR: Multidrug-resistant. Age ranges: Children, 0–11 years; Adolescent, 12–18 years; Young Adult, 19–24 years; Adults, 25 years and older.

Interviews lasted between 1 to 2 hours and were led by a team of highly experienced male and female bi-lingual researchers (MK, RNT, JC and AKH) from the Papua New Guinea Institute of Medical Research (PNGIMR). Interviews were conducted in a variety of languages including English, Tok Pisin (a lingua franca of PNG) and Kiwai (a local language) and were audio-recorded with informed consent from participants. Interviews took place in several locations including Daru General Hospital, the DART sites, offices, and some residential areas. The research team sought audio-confidential spaces to ensure privacy.

Datasets were transcribed and where necessary, translated to English, in preparation for analysis, by staff from PNGIMR.

### Data analysis

A thematic analysis approach was utilised for all data, whereby all data transcripts were coded in NVIVO 12 in a process of analytical induction, focusing on the identification of recurrent patterns [28]. Inductive thematic analysis resulted in a coding schema produced by two researchers (PJ and MK), and data was coded to themes related to experiences with counsellors and the education and counselling program, attributes of counsellors, and the counsellor’s role and responsibilities.

Coding of transcripts was cross-checked for consistency by research staff in PNG and Australia and revised considering emergent themes. Initial codes were analysed in detail to identify sub-themes as well as differences and similarities within and across the transcripts. This approach is iterative and reflexive, with each piece of data building on the next, to develop overall thematic concepts.

### Ethics

Ethics approval was received from the PNGIMR Institutional Review Board, the PNG National Department of Health’s Medical Research Advisory Committee, and UNSW Sydney (HC180602). The study was endorsed by the Western Provincial Health Authority in Papua New Guinea. All participants were informed of the study by key informants working in outpatient and inpatient medical services in the study sites and explained what would be required from them. Written consent forms were obtained from the study participants who were literate, while witnesses signed on behalf of those who were unable to read and write. Participants were also given the option of giving verbal consent if they were more comfortable doing so. Regarding the inclusion of interviews with people on treatment who were adolescents, two pathways to consent were sought: 1) parental or guardian informed consent was sought for the adolescent’s participation and 2) parents or guardians of adolescents were invited to participate and discuss the adolescent’s experience with TB treatment.

Authors did not have access to information that could identify individual participants during or after data collection. Pseudonyms have been used to protect participants’ identities.

## Findings

**Fig 1:**
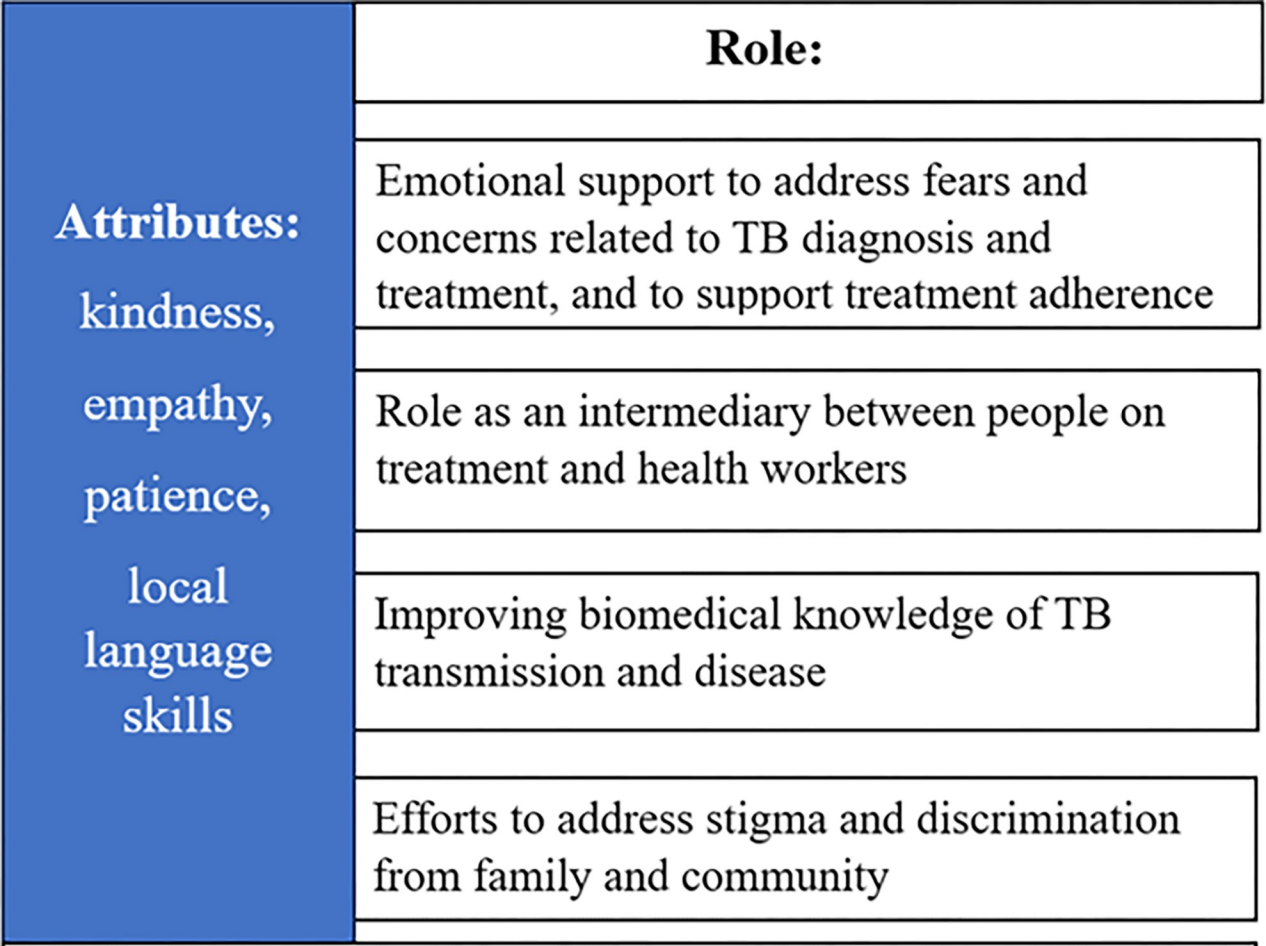
Overview of thematic concepts.

In the sections that follow, we highlight the main themes that emerged from the data analysis. The people interviewed spoke highly of the value of the counsellors and discussed the role they played in relation to the following themes: emotional support to address fears and concerns related to TB diagnosis and treatment, and to support treatment adherence; valuable attributes of counsellors; role as an intermediary between people on treatment and health workers; improving biomedical knowledge of TB transmission and disease; and efforts to address stigma and discrimination from family and community. Fig 1 gives an overview of thematic concepts.

The findings speak specifically to the experience of people on TB treatment with their counsellors and are primarily derived from interviews with people on treatment and/or caregivers and family members. However, it should be noted that key informants, such as health care workers and counsellors, also reflected positively on the themes identified. Their impressions of the value of counsellors are included. We also comment on the limits of an education and counselling approach and the impact of the role on counsellors.

### Emotional support to address fears and concerns related to TB diagnosis and treatment, and to support treatment adherence

Upon receiving a diagnosis of TB and being informed of the treatment plan, many participants spoke of their fears and concerns related to treatment duration and how this would affect their lives and families. Selina, a young woman diagnosed with MDR-TB at Daru Hospital, spoke of her sadness the day she received her diagnosis and was informed that treatment would include two years of daily medication, comprising eight months of injectable TB drugs. “Two years”, as she recalled, “it’s a really long time”. Selina was provided with a counsellor to support her through diagnosis and treatment. Selina said the counsellor provided her with encouragement and support to ease her fears, validating and exploring her emotional responses to issues as they arose, and providing biomedically accurate information as needed, including reaffirming that TB is curable. Selina said the counsellor assured her by saying, “Don’t feel scared. It’s for your own good that you must come to take your treatment…you will get better.” The counsellor stressed the importance of adhering to her treatment and Selina reported attending her local DART site every day since starting treatment:

I listen to them, and I used to come every day. Every 8 o’clock, I used to come here and take my medicine… because it’s good for me. Because I have a long life to live, I’m a young girl so I must come every day to take my treatment.

Rex, a young father of two, was diagnosed with MDR-TB, and said, “I was scared for the first few days I was taking medicine.” During the first two weeks on treatment Rex was taking 15 tablets daily with daily intramuscular injections, the latter which he described as painful. Initially reluctant to take the medicine, Rex said that the counsellor’s education and encouragement changed his mind: “I have to take that medicine because it’s for my own good and protects my family from getting TB. I have to be faithful to my medicine.”

According to Dr. Degba, a healthcare worker, “counsellors spend a lot of time encouraging patients to see beyond their infection.” Stevenson, a counsellor, believed that peer counsellors brought a unique understanding to the role because of their own experience with TB:

They have seen and experienced that you know, how TB affects the family and the lives of [patients]. These are the experiences they bring into the role as peer counsellors and then they support others. And this is very effective.

### Valuable attributes of counsellors

Counsellors were described as kind and empathetic listeners, with whom people could open up and share their worries. Theodore, an 18-year-old on treatment for DS-TB, said that he “felt better… felt motivated” because his counsellor “spoke to me very nicely.” This support and encouragement enabled Theodore to complete his DS-TB treatment while living with his uncle in Daru, away from the rest of his family (mother, father, and five siblings) who were back in his village on the mainland.

Robin, the mother of a child on TB preventive treatment, also spoke positively of the counsellors’ attributes. Counselling is important when parents and caregivers have to make the decision to put an otherwise healthy child on daily medication for three months for TB infection, when the dormant TB bacteria is already in their body but not causing symptoms. Robin described how having a counsellor to engage with during the treatment process made it easier:

The way [counsellors] talk, and every approach, facial [expressions] are very good and kind in a way that you can really understand… I’ve seen that it’s very good, where you can feel comfortable to express whatever you are hiding inside. They are trained for that… they are doing a great job.

When asked how the counsellors treated him, Taison, a person on treatment for TB, appreciated the counsellors’ empathy, “they are they are nice, some try to come to the level where they try to understand you.” Dr. Valentine, a key informant interviewed from Daru General Hospital, stated that the personal attributes of counsellors, such as their communication skills and patience, and the services they provide is one of the primary reasons the TB treatment program is so effective:

And I think it is a relationship as well; listening [to] them, talk to them, and supporting them and to solve their problem. It’s a really important program. I think this is one of the important components in Daru [and] that’s why you can see the success rate.

### Role as an intermediary between people on treatment and health workers

Counsellors were identified by participants as a good intermediary between people on treatment and doctors at Daru General Hospital and health workers at DART sites. Some people on treatment described feeling nervous when speaking directly to the health care workers overseeing their treatment about any concerns they had about their diagnosis or treatment. Mason, who was on treatment for MDR-TB, said, “they are very helpful [counsellors]…sometimes they use to talk on behalf of us [to the doctor] to support us.” Rita, also on treatment for MDR-TB, explained how the intermediary role worked:

[counsellors] put [in a] request for the doctors to see [me] if I complain on certain things that I didn’t want. The counsellors go and report [it] and then they send message to the treatment supporters which are in the DART site. And then we are requested to go and see the doctors and they take us up to see the doctors.

In Daru, several of the senior TB specialists providing high level technical and clinical support were from overseas. Cameron, a person with MDR-TB, stated that “sometimes people are scared to talk with the [international] doctors”, and felt more at ease talking with the Papua New Guinean counsellors who would then “talk with the doctors by explaining the patient’s sickness” on their behalf. Marvin, a caregiver of a child on preventive treatment, explained the importance of counsellors being able to speak a range of the local languages, making it possible for people to communicate in their first language. He said, “they [counsellors] come down to their [the person on treatment for TB] level and they use their mother tongue to speak to them.” Being treated as an equal was an important and valued dynamic for people on treatment and their families.

### Improving biomedical knowledge of TB transmission and disease

Participants felt strongly that counsellors played an important role in filling TB biomedical knowledge gaps related to transmission, prevention, and treatment. Thadelyn, a woman on treatment for MDR-TB, explained that counsellors “are the ones who are telling us about TB.” She detailed the type of information counsellors provided to people with TB to prevent onward transmission of the infection:

If you do not want to get sick, you don’t sleep in a crowded house… If you are sick and you are coughing you must not open your mouth and cough to your families… stand away from them so the TB will not spread.

Kono and her 11-year-old son were both on treatment for MDR-TB. They relocated to Daru from a village in the Middle Fly District of Western Province for two years of treatment and were put in contact with a counsellor immediately. Kono liked the visual tools counsellors used to explain the difference between TB infection and disease, and a book they used to describe how to reduce transmission risks and the importance of treatment adherence:

They opened it [the book]…okay, if you will stay in the house, okay no windows, and that inside is no air, okay then you will get this sickness…If you will get your medicine good okay you will be better.

The importance of the educational role of counsellors was reiterated and reinforced by TB healthcare workers and other key informants such as TB program implementers. One healthcare worker said that counsellors were “empowering” for people on treatment, encouraging “them to become advocates for TB education” in their own communities.

### Efforts to address stigma and discrimination from family and community

While not pervasive, TB-related discrimination and stigma was reported by some people in our study, some of which was related to limited biomedical knowledge. This was the experience of Sharon, a former TB patient. At the time of our interview, Sharon’s son, Felana, was receiving TB treatment in Daru. Sharon was from the mainland South Fly District but living in Daru with extended family so Felana could get treatment. Sharon experienced problems with family members as they were afraid Felana was contagious, even though he was on treatment. They did not want to share plates and utensils with Felana and indicated that Felana and Sharon should move out. Sharon spoke with Felana’s counsellor to gather biomedical information about TB to address these issues with her family:

I got all that information [from the counsellor] and I went to the house and told them [family] about it. [I said], “You people want [me to] bring the counsellor to you guys and then she will sit you guys and explain everything to you people? Why are you people feeling scared of him? He is on treatment… I told them, “TB cannot pass from plates and spoons, even sleeping together [and] even sharing clothes.”

Sharon appreciated that the counsellors answered all her questions related to TB transmission, so she was well-informed to counter any false beliefs or stigma from her family.

Following his diagnosis, Brad, a young man with MDR-TB, was worried what people in Daru would think of him. He told us that, while his family was supportive and welcomed TB education visits from his counsellor, he worried about the reactions from the wider community. Brad spoke of the encouragement he received from counsellors and how they helped him cope with this:

[They] tell the patients for them to not feel down because discrimination too is going [happening] and encouraging them that, “it’s alright, you are alright… don’t give up, continue coming and getting medicine.”

While they saw the benefits of counselling and counsellors, two participants mentioned that they would have liked more privacy when speaking to their counsellor as they were worried about being noticed by other people. As Taison, a person on treatment stated, “it’s quite open [where counselling takes place] and I sort of wanted to avoid the picture where people might see me.” He asked if he could speak to his counsellor in a more private office at the DART site, and the counselling team arranged this.

Sally, a TB healthcare worker, felt that TB education and awareness with families and the wider Daru community had significantly reduced stigma and discrimination and was a testament to the strengths of the education and counselling program:

Our counsellors made an attempt to step in for our patients. When they were stigmatised and all that discrimination going on…we had to talk to people who were criticising our patients, so now it doesn’t happen anymore.

### Limits of the approach and impact on counsellors

While the aim of this article was to examine how counsellors enhanced the treatment experience of people on treatment for TB disease and for caregivers of children on TB preventive treatment, it is also important to mention the limits of the education and counselling approach and the effect the role had on the counsellors themselves. As noted in the previous sections, counsellors provided TB education; however, people on treatment, caregivers, and counsellors themselves noted the limits of TB education-based approaches, which were not able to overcome the structural influences on TB transmission in Daru. Clement, a caregiver of a child on treatment for DS-TB, said that the counsellor advised her “how to fix our living standard in the community, in the house, how to get fresh air, maintain cleanliness and about the people in the community”. When asked by the interviewer what changes she had made since receiving this advice, she said,

the yard too is overpopulated and with regard to cleaning the house it is also overpopulated with people so I didn’t bother to clean the house, but I am only cleaning the yard (*laughing*) as it is hard to move the people.

Here Clement alludes to the wider socio-structural issues in Daru associated with being overpopulated and overcrowded, and the increasing pressure on housing space as more family members relocate to Daru from mainland South Fly District to access centralised health services. The counsellor gave advice on how to reduce risks of TB transmission, but Clement outlined real life experiences that hindered such mitigation efforts. These challenges were confirmed by staff within the education and counselling program. Victoria, a key informant from the program, explained:

Another challenge… anyone who starts in counselling is dealing with situations where there aren’t any resolutions for patients. There’s kind of chronic issues I was talking about before, like lack of accommodation or lack of food and so of course our counsellors are all very compassionate and supportive people, and they want to try and assist the patients to the best solutions possible for them, so it’s very hard for them. Many situations that can’t be resolved by counselling, which is plenty.

Counsellors played an important role in the lives of people on treatment and their families and while they wanted to find the best solutions for the people they engaged with, they were aware that, as Victoria mentioned, education and counselling cannot resolve all issues, and also of the emotional toll this could have on their own mental health. As Vero, a counsellor noted:

We have the difficult cases where you are dealing with peoples’ minds and sometimes you go home, you take that loads with you… We need some days [to take] a break just to unwind all these loads we have had because we are listening and listen to many, some we absorb, some is stressed into us and we take it back on our families too. Being a counsellor, I am listening, and I am absorbing so many things from other people into my space.

Counsellor Stevenson commented on the personal impact some of his counselling cases have had on him:

The lives we are dealing with and when we see those kinds of challenges our people are going through, it’s really bad…. I have to go for supervision so that I am not burdened with what I have seen and what I experienced every day.

To address their own self-care and avoid emotional burn-out, the counsellors were offered self-care days, daily debriefings with senior staff, and ‘External Supervision’ once a month with their supervisor and other counsellors so they could talk about situations that were impacting them. The counsellors interviewed were passionate about their jobs, but they recognised that they were at risk from emotional burnout and therefore appreciated the offer and recognised the importance of supervision and a focus on their own mental health.

## Discussion

Our findings indicate that people on treatment for TB, as well as their caregivers and family, had a positive experience with counsellors and the services they provided, and felt supported throughout their treatment journey. Counsellors were perceived as a valuable source of social and emotional support throughout treatment, allowing people to speak candidly of their fears and concerns upon receiving their TB diagnosis, accepting that treatment would be a long-term commitment, and processing how diagnosis and treatment would affect their daily life. Such benefits of support have been found in other international studies [4,29,30]. Participants described how counsellors also supported them through to completion of treatment. Our findings concur with other studies illustrating how education and counselling can improve treatment adherence for TB disease [7,12,19,31] and TB infection, known as TB preventive treatment [19,20]. Peer support, education and counselling has also been found to play an essential role for people living with HIV [32] and is recommended by the WHO for adolescents and young people living with HIV [33].

Rather than assuming anyone (e.g., health workers) can “counsel” and give good education to people on treatment, our findings illustrated specific attributes of the counsellors that were valued by people in treatment, making them feel understood and heard. These included kindness, empathy, patience, local language skills, and a specific demeanor, approach and communication style that can be developed through training and practice, along with the time needed to build relationships that create trust. The importance of these attributes should not be undervalued. Studies have shown that the attitude and demeanor of health care workers and people who provide TB services can impact on the person with TB’s motivation to complete treatment [34,35].

The perceived value of the education component of the counsellors’ work in Daru was also clear, enhancing knowledge and understanding of TB transmission, prevention, and treatment. This was appreciated at an individual level by people we interviewed, but benefits also extended to caregivers, families, and wider communities, related to stopping TB transmission and addressing stigmatisation and discrimination. It has been reported elsewhere that individual patient counselling, along with health education for the TB patient’s family and community, can be an effective intervention to address stigma [36,37].

Participants also noted the limits of an education and counselling approach due to the complex needs arising from their current living situation and the constraints associated with Daru being the only place in the South Fly District where people can be diagnosed and treated for MDR-TB. The counsellors practice an empowerment approach where the goal is to provide people with TB with emotional and informational support and help them navigate and find solutions to the personal, familial and health system challenges associated with TB diagnosis and treatment [38]. The findings note many issues experienced by people on treatment and their families–overcrowded households, financial struggles, relocation–that cannot be resolved by education and counselling. This is likely best dealt with through a more decentralized health system enabling diagnosis and treatment closer to home.

### Limitations

The examination of the education and counselling program was a small component of the overall study. Questions were semi-structured, meaning that participants spoke only to the issues they perceived as important to them, which limited the depth of questioning about this work and missed opportunities to talk to particular dynamics of the counselling process. Key areas we would like to know more about include the benefits and constraints of the ‘peer’ approach to counselling, as well as a deeper understanding of the lived experiences of the counsellors themselves.

### Implications for policy and practice

Despite these limitations, this is one of a limited number of qualitative studies related to TB in PNG [25,39–41]. Formally collected and reported evidence that has informed the TB response in PNG has been largely clinical, biological or epidemiological in nature [24,42–47], and this study integrates a social dimension to this approach, a potentially critical aspect which to date has been largely neglected in PNG and elsewhere. It adds to evidence supporting TB education and counselling initiatives as key components of TB care [19,21,24]. Our analysis points to several practical lessons. First, as countries start to implement shorter, safer treatment regimens for TB disease and infection which promote models of decentralized and community-based care, it will be critical to provide education and counselling to support the complex challenges of adherence that will exist irrespective of regimen length. This is particularly important for TB preventive treatment, which is often implemented in a facility-based model and has more challenges in providing education and informational support for treatment acceptance and adherence. Second, it would be useful to continue scoping out the full range of responsibilities, skills and interpersonal attributes of counsellors in PNG and other settings, to identify where they best fit within the cascade of health workers and to support the advantages of peer approaches in TB elimination efforts. Third, when thinking about the future of person-centred TB care in complex socio-cultural and health system settings like PNG, it will be important to assess how to integrate education and counselling into health system strengthening work, where services become more decentralized and less facility-based for treatment.

## Conclusion

Education and counselling is strongly recommended by the WHO for people on TB treatment. Our findings provide strong evidence that counsellors, and in particular peer counsellors, play an essential role in TB treatment services in PNG, helping alleviate stressors that compound the overall physical stress of TB treatment, and providing people with the encouragement to continue treatment to completion while feeling supported and empowered. The findings build on global evidence that education and counselling is an important component of TB care.

## Supporting information

S1 Checklist (DOCX)
