# Supplementary material for: The role and value of counsellors in the treatment journeys of people with tuberculosis and their families: Qualitative insights from the South Fly District of Papua New Guinea
Source: PLOS Glob Public Health. 2024 Oct 21;4(10):e0002572. doi: 10.1371/journal.pgph.0002572 (PMC11493273; doi:10.1371/journal.pgph.0002572)
Supplement: S1 Checklist — (DOCX) [file pgph.0002572.s001.docx]

Inclusivity in global research

PLOS’ policy on inclusivity in global research aims to improve transparency in the reporting of research performed outside of researchers’ own country or community and ensures that PLOS publications reporting global research adhere to high standards for research ethics and authorship. Authors of relevant research articles may be asked to complete the questionnaire below, which outlines ethical, cultural, and scientific considerations specific to inclusivity in global research. This questionnaire may be requested when researchers have travelled to a different country to conduct research, if research uses samples collected in another country, research with Indigenous populations or their lands, or if research is on cultural artefacts. Researchers travelling to another country solely to use laboratory equipment will not normally be required to complete the questionnaire. However, the questionnaire can be requested at the journal’s discretion for any submission – if you have been requested to complete this questionnaire by the PLOS journal you submitted to, please do so.

Please complete the questionnaire below and include this as a Supporting Information file with your manuscript. Note that if your paper is accepted for publication, this checklist will be published with your article in the supporting information files. Please ensure that you reference the checklist in the main body of your manuscript. We suggest adding a subsection ‘Inclusivity in global research’ to your Methods section and adding the following sentence: “Additional information regarding the ethical, cultural, and scientific considerations specific to inclusivity in global research is included in the Supporting Information (SX Checklist)”

The questions have been designed to be applicable to a wide range of study types, and there are subsections for both human subjects research and non-human subjects research. If any of the questions are not relevant to your research please mark them as “N/A” as appropriate.

**Ethical considerations, permits and authorship**

*This section is applicable to all research types.*

Provide details as to who granted permissions and/or consent for the study to take place in the Methods section of your manuscript. This should include the names of **all** ethics boards, governmental organizations, community leaders or other bodies that provided approval for the study. If individuals provided approval refer to these people by their role or title but do not list their name(s).

Reported on page number: 8

If there were any deviations from the study protocol after approval was obtained please provide details of these changes in the Methods section of your manuscript.
Did this study involve local collaborators that are residents of the country where the research was conducted or members of the community studied? If you do not have any authors from said communities, please provide an explanation for this below.

Reported on page number: N/A

A national advisory group was established in order to design the study in consultation with a diverse group of interested parties in Papua New Guinea. This included representatives from the National and Provincial Departments of Health and Health Authorities. Key personnel from the Papua New Guinea Institute of Medical Research (PNGIMR) were consulted, and two staff members from PNGIMR are also involved as AI’s on this study. The PNGIMR and UNSW jointly led the study. Stakeholder meetings continued throughout the duration of the study and included key NDoH personnel in PNG’s TB response. Local advisory groups were also established in the study site. Consultative stakeholder meetings and community workshops were conducted prior to the start of the study, with the aim of identifying and initiating local community advisory groups consisting of people living with TB, health care workers and community representatives, including in-country CIs. Fieldwork staff on our study were all residents of Papua New Guinea, and staff from Papua New Guinea and Australia were involved in analysis of study data.

Additionally, seven of the authors on our paper are from Papua New Guinea and continue to reside there.

Everyone listed as an author should meet PLOS’ criteria for authorship and all individuals who meet these criteria should be included in the author byline, rather than the acknowledgements. For further information please see the journal’s Authorship Policy.

**Human subjects research (e.g. health research, medical research, cross-cultural psychology)**

Did you obtain written informed consent from a representative of the local community or region before the research took place? How did you establish who speaks for the community? Details of written informed consent obtained from study participants should be reported separately in the Methods section of your manuscript.

The research site was chosen in partnership with the National Department of Health in Papua New Guinea. Ethics approval for the study protocol was received from the Papua New Guinea Insitutue of Medical Research (PNGIMR) Institutional Review Board, the PNG National Department of Health’s Medical Research Advisory Committee, and UNSW Sydney (HC180602). The study was also endorsed by the Western Provincial Health Authority in Papua New Guinea. Please note, our study site (South Fly District) is located within the Western Province of Papua New Guinea.

Details of written informed consent obtained from study participants is detailed in the ‘Ethics’ section of our manuscript.

How did members of the local community provide input on the aims of the research investigation, its methodology, and its anticipated outcome(s)?

When engaging with the local community, how did you ensure that the informed consent documents and other materials could be understood by local stakeholders?

Stakeholder and community engagement was led by a team of highly experienced male and female bi-lingual (English and Tok Pisin) researchers from PNGIMR. Documents were also translated into local language. In a few cases where people spoke the local languages in South Fly District (Kiwai), the team was supported by a locally engaged speaker.

As mentioned, local advisory groups were established in the study sites. Consultative stakeholder meetings and community workshops were conducted prior to the start of the study, with the aim of identifying and initiating local community advisory groups consisting of people living with TB, health care workers and community representatives, including in-country CIs. During these meetings and workshops we gave participants the opportunity to comment on the study aims, objectives, and methodology.

The interview guides were developed in consultation with local organisations involved in the national and sub-national response to TB and the study’s Advisory Groups.

Will the findings of the research be made available in an understandable format to stakeholders in the community where the study was conducted (e.g. via a presentation, summary report, copies of publications, etc.)? Please provide details of how this will be achieved.

Yes. In March 2023 members of the research team from Papua New Guinea and Australia held a dissemination meeting with key stakeholders in Papua New Guinea that included the Western Provincial Health Authority, the Daru Mayor and his staff, the South Fly District Administrator and senior staff, staff from the Burnet Institute in Daru, and members of the South Fly District Development Authority. We also made presentations to community members with the study findings.

**Non-human subjects research using specimens/ animals collected as part of the study, or those housed in archival collections. Examples include archaeology, paleontology, botany and zoology.**

Did the permission you obtained from a local authority to perform the study include an agreement on access to outputs and benefit sharing? This may include procedures to enable fair distribution of the benefits and resources arising from the research performed. Please include any details of Prior Informed Consent and Benefit Sharing Agreements obtained. These may be required by field-specific regulations, for example the Convention on Biological Diversity (CBD) and the associated Nagoya Protocol.

N/A

If the material used in your study was imported, please A) provide the year it was imported and B) indicate whether permits were obtained to import/export the materials used, C) provide details of any permits obtained. If this information is not available, please indicate this.

N/A

If you used archival specimens, please state how the material used in your study was acquired by the institute it is held in and provide details of any permits obtained for the original excavations/ sample collection. If this information is not available, please indicate this.

N/A

How was the potential cultural significance of the materials collected in your study to local communities considered in your research design? Were Indigenous peoples and/or local researchers and institutions involved with archaeological excavations / collection of specimens? If so, please provide a description of their involvement.

N/A

If your manuscript includes photographs of human remains please indicate whether authors obtained permission from descendants or affiliated cultural communities to do so.

N/A
